# Zoonotic Bacteria in *Anolis* sp., an Invasive Species Introduced to the Canary Islands (Spain)

**DOI:** 10.3390/ani13030414

**Published:** 2023-01-26

**Authors:** Néstor Abreu-Acosta, Román Pino-Vera, Elena Izquierdo-Rodríguez, Oscar Afonso, Pilar Foronda

**Affiliations:** 1 Nertalab S.L.U. Santa Cruz de Tenerife, Tenerife, 38001 Canary Islands, Spain; 2 Instituto Universitario de Enfermedades Tropicales y Salud Pública de Canarias, Universidad de La Laguna, La Laguna, Tenerife, 38200 Canary Islands, Spain; 3 Department Obstetricia y Ginecología, Pediatría, Medicina Preventiva y Salud Pública, Toxicología, Medicina Legal y Forense y Parasitología, Universidad de La Laguna, La Laguna, Tenerife, 38200 Canary Islands, Spain; 4 Área de Medio Ambiente, Gestión y Planeamiento Territorial y Ambiental (Gesplan), Santa Cruz de Tenerife, Tenerife, 38200 Canary Islands, Spain

**Keywords:** *Anolis* sp., Canary Islands, zoonotic bacteria

## Abstract

**Simple Summary:**

The anoles are a group of lizards native to America that have been introduced to other regions, such as the Canary Islands (Spain). With this study we aimed to analyze the presence of pathogenic bacteria in a population of this invasive lizard in the Canary Islands. The results highlight the presence of a variety of pathogens of relevance to health, most of them related to gastrointestinal diseases. This archipelago is considered a hotspot of biodiversity, where some species are considered endangered, so the presence of anoles can also be a risk for the biodiversity conservation, by the spread and/or transmission of pathogenic bacteria to the native fauna. In conclusion, the invasive anoles population in the Canary Islands should be considered as a potential risk factor for public health and biodiversity conservation.

**Abstract:**

Lizards belonging to the genus *Anolis* are native to America and have been introduced in many parts of the world. In this work, a gastrointestinal microbiological analysis from *Anolis* sp. introduced to Tenerife, Canary Island, was carried out. A total of 74 individuals were analyzed by culture and molecular tools. *Pseudomonas* spp. was the most prevalent bacteria isolated (64.3%), followed by enteropathogenic *Escherichia coli* with at least one of the investigated virulent genes (*stx_1_, stx_2_,* and *eae*) (44.6%). The *stx_2_* gene was more prevalent which differs to that reported in other reptiles, probably due to wastewater transmission. *Campylobacter* spp. was detected in 32.4% of the animals, highlighting the detection of *C. jejuni* and *C. fetus* by their relevance to public health. The zoonotic *Staphylococcus lugdunensis*, found in 14.9% of the animals, was firstly detected in reptiles. *Vibrio* sp. which is more associated with aquatic environments was found in 10.8% of the lizards in this study, with *Vibrio cholerae* being found in two of the animals. The prevalence of *Salmonella* sp. (5.4%) was low, compared with other studies carried out in reptiles. These results indicate that *Anolis* sp. in Tenerife could be playing a role in the maintenance and spread of the pathogens detected, being a possible risk factor for public health and biodiversity conservation.

## 1. Introduction

Following the description of the Convention on Biological Diversity (CBD, 2010), invasive non-native species (INNS) are organisms introduced outside their natural distribution area due to human activity, or are species that can threaten biodiversity, being one of the largest global threats to biodiversity (IPBES global assessment, 2019). Invasions are not the result of single species introduction events, but units of biological organization that include the host and all its symbionts, including pathogenic species [1]. Therefore, invasive species can constitute a health risk for humans, animals, and plants, acting as reservoirs for pathogens [2]. Island communities are more vulnerable to introduced diseases because of their geographical isolation [3,4]. While the impact of foreign diseases on the human communities of the islands is well-documented [5], the effect of introduced diseases on biodiversity is less reported [6]. However, there are evident examples of introduced diseases that are seriously affecting the survival of island species [7].

The Canary Archipelago (Spain), located in NW Africa (13°23′–18°8′ W and 27°37′–29°24′ N), is a particularly suitable place for the settlement of invasive species due to its climatic conditions, its wealth of resources, and the lack of large predators that control the populations [8]. Tenerife, the largest island in the Archipelago, is also the island with the largest number of exotic species. According to the latest available data, around 1200 of the species present are not native to the island, and a significant percentage of these could become naturalized and behave in an invasive manner, affecting the island biodiversity [9]. Among the invasive species, lizards belonging to the genus *Anolis* Daudin, 1802 (Squamata, Dactyloidae) were introduced in the south of the island through the ornamental plant trade since 1995 [10]. These small tree lizards are native to North America, but they have been introduced to many parts of the world [11,12,13], harming the ecosystems of some regions, as in the case of Chichi Island, Japan [14].

Due to the lack of data on the presence of pathogenic micro-organisms in this invasive *Anolis* species in the Canary Islands, the main purpose of this study was to determine the presence of pathogenic bacteria in these lizards and to evaluate the possible health risks for public health and native fauna.

## 2. Materials and Methods

A total of 74 animals, 70 adults (25 males and 45 females) and 4 young individuals were captured manually and by adhesive traps in the South of Tenerife (Canary Islands, Spain). The captured anoles were euthanized after authorization of “Dirección General de Lucha Contra el Cambio Climático y Medio Ambiente” (Gobierno de Canarias) (Expte. 31280/2021). The animals were measured and weighed, and then necropsied and their sex determined. Fecal samples were obtained during the dissection for culture.

### 2.1. Bacterial Strains

The bacterial strains used as positive amplification controls were obtained from American Type Culture Collection (ATCC). All the bacterial strains were stored at −70 °C and were grown on Tryptic Soy Broth (TSB; Labkem, Barcelona, Spain) at 37 °C for 18 to 24 h under aerobic or microarophilic conditions, in the case of *Campylobacter* spp. Sebald and Veron, 1963; Vandamme et al., 2010.

### 2.2. Isolation of Pathogenic Bacteria

For the investigated bacteria, except for *Campylobacter* sp. and *Vibrio* spp. Pacini, 1854, 0.1 g of intestinal content was extracted and incubated (37 °C) in 5 mL of Buffered Peptone Water (BPW) (Labkem, Barcelona, Spain) for 24 h thus achieving enriched liquid culture. In the case of *Campylobacter* sp., 0.1 g of the intestinal content was taken in a 2 mL tube with BPW. The tubes were incubated at 42 °C for 18 h in microaerophilic condition. For *Vibrio* spp., 0.1 g of intestinal content was transferred into 5 mL of Alkaline Peptone Water (APW) containing 1% NaCl as a pre-enrichment, incubated for 8 h at 37 °C.

After the incubation, for the isolation of *Salmonella* spp. Lignieres, 1900, 0.5 mL of each culture of BPW was transferred into 4.5 mL of Rappaport Vassiliadis Broth (VWR International, Leuven, Belgium) and incubated a 42 °C for 20 h. Then, these samples were cultured on *Salmonella-Shigella* agar (Merck, Darmstadt, Germany) at 37 °C as a secondary enrichment culture. For the isolation of the other pathogenic bacteria considered in this study, 100 µL of each culture of BPW were inoculated in selective media for *Staphylococcus* sp. Rosenbach, 1884 (Baird Parker agar; Labkem, Barcelona, Spain), *Pseudomonas* sp. Migula, 1894; Yang et al., 2013 (Cetrimide agar; VWR International, Leuven, Belgium), and *Listeria monocytogenes* (Murray et al., 1926) Pirie, 1940 (Oxford agar; Labkem, Barcelona, Spain). In the case of *Vibrio* spp., 100 µL of each culture of APW were inoculated onto Thiosulfate–Citrate–Bile salts Sucrose agar (TCBS; VWR International, Leuven, Belgium) and incubated for 24 h at 37 °C.

### 2.3. Molecular Identification of Isolates and Pathogenicity Genes

#### 2.3.1. DNA Isolation

In the case of *Campylobacter* spp., 1 mL of BPW incubated at 42 °C, and in the case of *Escherichia. coli* (Migula, 1895) Castellani and Chalmers, 1919, *Mycobacterium* spp. Lehmann and Neumann, 1896; Gupta et al., 2018, and *Yersinia enterocolitica* (Schleifstein and Coleman, 1939) Frederiksen, 1964, 1 mL of BPW incubated at 37 °C were taken, respectively. They were washed twice with PBS and the pellet was subjected to DNA extraction according to López et al. [15].

For the rest of the bacteria analyzed, 5 colonies of each culture were dissolved in 1 mL of PBS and centrifuged at 12,000× *g*. The supernatant was removed and centrifuged again under the same conditions. The pellet was subjected to DNA extraction following López et al. [15].

#### 2.3.2. PCR Assays

A multiplex-PCR (m-PCR) assay was used for the identification of the most important zoonotic *Campylobacter* species, *C. jejuni* (Jones et al., 1931) Veron and Chatelain, 1973, *C. coli* (Doyle 1948) Veron and Chatelain, 1973, *C. lari* Benjamin et al., 1984; Debruyne et al., 2009, *C. upsaliensis* Sandstedt and Ursing, 1991, and y *C. fetus* (Smith and Taylor, 1919) Sebald and Veron, 1963, according to the specifications of Wang et al. [16].

Enteropathogenic *E. coli* (producing shigatoxins, STEC) were analyzed by the detection of the genes that encode for Shiga toxins (*stx1* and *stx2*) and the gene that codes for the intimin protein, which is another factor associated with virulence in *E. coli*, following the protocol described by Blanco et al. [17].

Other m-PCR assay was carried out for the identification of *Mycobacterium* sp. and, in addition, for the differentiation of the species belonging to *M. tuberculosis* complex, according to Kim et al. [18].

For the identification of *Yersinia enterocolitica*, a duplex PCR assay described by Wannet et al. [19] was developed. With this protocol, non-virulent and pathogenic strains are detected.

Other PCR assay was used for the detection and identification of *Vibrio* spp. according to Liu et al. [20]. The samples that were positive were subjected to an m-PCR assay according to Neogi et al. [21], for the identification of *Vibrio cholerae* Pacini, 1854, *V. parahaemolyticus* (Fujino et al., 1951) Sakazaki et al., 1963; West et al., 1986, and *V. vulnificus* (Reichelt et al., 1979) Farmer, 1980; West et al., 1986.

For the identification of the most prevalent zoonotic *Salmonella* serotypes, two m-PCR assays were used, according to Guimarães de Freitas et al. [22]. In a first test, all *Salmonella* species are detected, in addition to *Salmonella* ser. Enteritidis and *Salmonella* ser. Typhi. In the second trial, *Salmonella* ser. Typhimurium is identified.

One m-PCR was used for the detection of clinically relevant antibiotic resistance genes harbored by some *Staphylococcus aureus* Rosenbach, 1884 isolates, and for the simultaneous identification of these isolates to the species level, according to Pérez-Roth et al. [23].

An m-PCR assay was used for the detection of fluorescent pseudomonads and the identification of *Pseudomonas aeruginosa* (Schroeter, 1872) Migula, 1900 based on the simultaneous amplification of the *oprI* and *oprL* genes, as described by De Vos et al. [24].

To confirm suspicious colonies grown on Oxford agar as *Listeria monocytogenes*, a region of the ***iap*** gene was amplified by PCR following the protocol described by Jaton et al. [25].

Positive and negative controls were included in all the PCRs performed.

After PCR amplification, 5 µL was analyzed in an agarose gel (Fisher Bioreagents, Madrid, Spain) electrophoresis to estimate the sizes of the amplification products by comparison with a molecular size standard ladder (GeneRuler 50 bp DNA Ladder, Thermo Scientific, Vilnius, Lithuania and HyperLadder 50 bp, Bioline, London, England). The gel was stained with Real-Safe (Durviz SL, Valencia, Spain), and the amplicons were visualized with ChemiDoc™ XRS+ (Bio-Rad, Hercules, CA, USA) system. Some amplicons were purified using the UltraClean PCR Clean-up (MO BIO Laboratories, Inc., Carlsbad, CA, USA) kit, following manufacturer recommendations. Sequencing in both directions was performed in Macrogen (Spain) and then analyzed with software MEGA X (Molecular Evolutionary Genetic Analysis) [26]. Sequences were compared and similarity values obtained with available published sequences in GenBank by using the nucleotide–nucleotide BLAST (blastn) program [27].

### 2.4. Index Co-Infection (Ic)

Ginsberg, H.S. [28] developed an index of co-infection (Ic) which quantifies the degree of departure of the number of mixed infections from independence. This is defined as the difference of the number of co-infections from the number expected due to chance alone, as a percentage of the total number of infected lizards.

Ic = [(O − E)/N] × 100,

where O = number of observed co-infections, E = expected number of co-infected ***Anolis*** sp. due to chance alone, and N = total number of ***Anolis*** sp. infected by either or both micro-organisms.

E = (a + b) (a + c)/(a + b + c + d),

N = a + b + c,

where a = number of lizards infected with both bacteria (equals O), b = number of *Anolis* sp. infected only with micro-organism 1, c = number of *Anolis* sp. infected only with micro-organism 2, and d = number of lizards not infected with either micro-organism. Ic is positive when the number of co-infections is greater than expected, and negative when there are fewer co-infections than would be expected due to chance alone.

Significance of the index was calculated by a chi-square test.

### 2.5. Statistical Analysis

A chi-square test, setting the *p* value in 0.05, was conducted to compare prevalence between age and sex using SPSS for Windows statistical software (IBM Corporation, Armonk, NY, USA).

## 3. Results

Seventy-four individuals were analyzed. Table 1 shows the percentages of isolation of the investigated bacteria. *Pseudomonas* spp. was the most prevalent bacteria (64.3%), followed by STEC with at least one of the investigated genes (45.6%), and *Campylobacter* spp. (32.4%).

### 3.1. Campylobacter spp.

In 24 individuals out of 74, *Campylobacter* species were detected by PCR, which represents a prevalence of 32.4%. No significant differences were found with the Chi-square test, between the number of positive male individuals (6/25; 24.0% of males) and females (16/45; 35.6% of females), or between positive adults and positive juveniles (2/4; 50.0% of positives) (Table 2). The length and weight of the animals that were infected with *Campylobacter* spp. were similar to those individuals that were free of these bacteria.

Four species were identified, with *C. fetus* being the most prevalent species, and the only one that was isolated in juvenile individuals. The second most prevalent species was *C. coli*, isolated only in females. The presence of *C. jejuni* and *C. upsaliensis* was also detected (Table 2). Moreover, non-identified *Campylobacter* species were detected in seven animals.

### 3.2. Vibrio spp.

Eight of the reptiles (10.8%) were positive for *Vibrio* sp., six of them females (13.3% of the females) and two males (8.0% of the males). The juveniles analyzed were free of *Vibrio* spp. The number of positives was statistically (*p* > 0.05) similar between males and females. The length and weight of the animals that presented *Vibrio* spp. were similar to those individuals that were free of these bacteria. Due to the infrequent detection of *Vibrio* spp. in terrestrial reptiles, the amplicons obtained in the PCR assay were sequenced. In two individuals, one male and one female, *Vibrio cholerae* was identified. Two of the obtained sequences had 100% similarity with *Vibrio cholerae* and were uploaded to the GenBank database (accession number OP688106 and OP688107).

### 3.3. Enteropathogenic Shiga Toxin Producing Escherichia coli (STEC)

**Figure 1:**
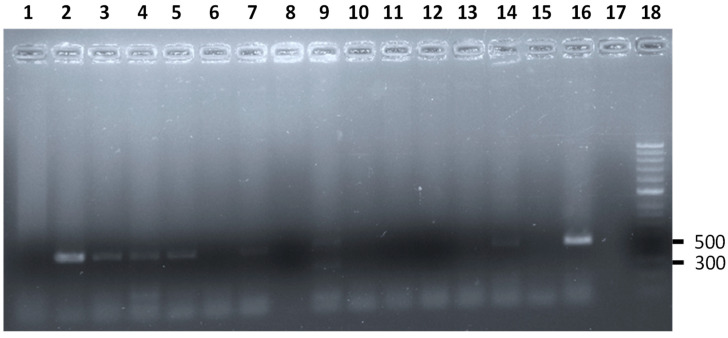
Results of the m-PCR assay for the simultaneous detection of *stx_1_* and *stx_2_* genes of *E. coli* STEC from *Anolis* sp. analyzed. Lane 2, 3, 4, 5, 7, and 14 amplification fragments of the *stx_2_* gene characteristic of *E. coli* STEC obtained from *Anolis* sp. analyzed in this study (516 bp). Lane 9 amplification fragments of the *stx_1_* (302 bp) and *stx_2_* gene characteristic of *E. coli* STEC obtained from one *Anolis* sp. analyzed in this study. Lane 1, 6, 8, and 15 *Anolis* sp. negative for *E. coli* STEC. Lane 16, positive control *stx_2_* gene. Lane 17, control negative. Lane 18, molecular size marker (HyperLadderTM 50 bp, Bioline).

The presence of Shiga toxin (*stx_1_*, *stx_2_*) and intimin (*eae*) genes, related to the ability of enteropathogenic *Escherichia coli* to produce disease, was analyzed (Figure 1). *E. coli* carrying these genes were found in 33 individuals (44.6%). The prevalence of STEC that carried the *stx_2_* gene (28.4%) was higher than that of those that carried the *stx_1_* gene (10.8%) and the *eae* gene (5.4%) (Table 3) (*p* < 0.05). There were no differences between the number of positive males and females or between adults and juveniles for the different virulence genes (Table 3). Finally, the coexistence of two virulence genes (*stx_1_* and *stx_2_*) was detected in two individuals, one female and one male.

### 3.4. Salmonella spp.

**Figure 2:**
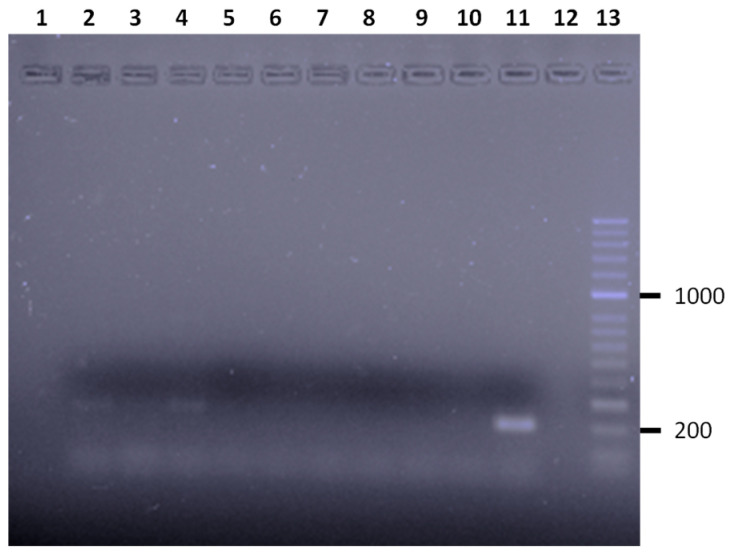
Results of the m-PCR assay for the simultaneous detection of *Salmonella* spp. and *S. enteritidis*, *S. typhimurium*, and *S. typhi* from various *Anolis* sp. analyzed. Lane 2, 3, and 4, amplification fragment of the *ompC* gene characteristic of *Salmonella* spp. obtained from *Anolis* sp. analyzed in this study. Lane 5 to 10, *Anolis* sp. negative for *Salmonella* spp. Lane 11, positive control. Lane 12, control negative. Lane 9, molecular size marker (HyperLadderTM 50 bp, Bioline).

The presence of *Salmonella* spp. was revealed by obtaining a 204 bp amplification fragment corresponding to the *ompC* gene, responsible for the C protein involved in the invasion of the epithelial cells of bacteria of the *Salmonella* genus, in four individuals (5.4%) of the total analyzed (Figure 2).

Two females were positive for *Salmonella* spp. (4.4%; n = 45), one juvenile (25.0%; n = 4), and one male (4.0%; n = 25). The investigated zoonotic serotypes (*Salmonella enteritidis*, *S. typhimurium,* and *S. typhi*) were not detected, since the expected amplification fragments of the *sdfI*, *spy,* and *viab* genes, respectively, were not obtained.

### 3.5. Staphylococcus sp.

Of the six species of this genus investigated, only *Staphylococcus lugdunensis* Freney et al. 1988 was detected, with a prevalence of 14.9% (11 positive/74 individuals).

The size of *Anolis* sp. individuals positive for this bacteria genus was similar to that of those in which these bacteria were not detected. However, the anoles that contained *Staphylococcus lugdunensis* had significantly lower weight ($\bar{X}$ = 4.36 g; Des. St = 0.57) than those individuals that were free of these bacteria ($\bar{X}$ = 5.76 g; Des. St = 2.25) (*p* < 0.05).

### 3.6. Pseudomonas spp.

**Figure 3:**
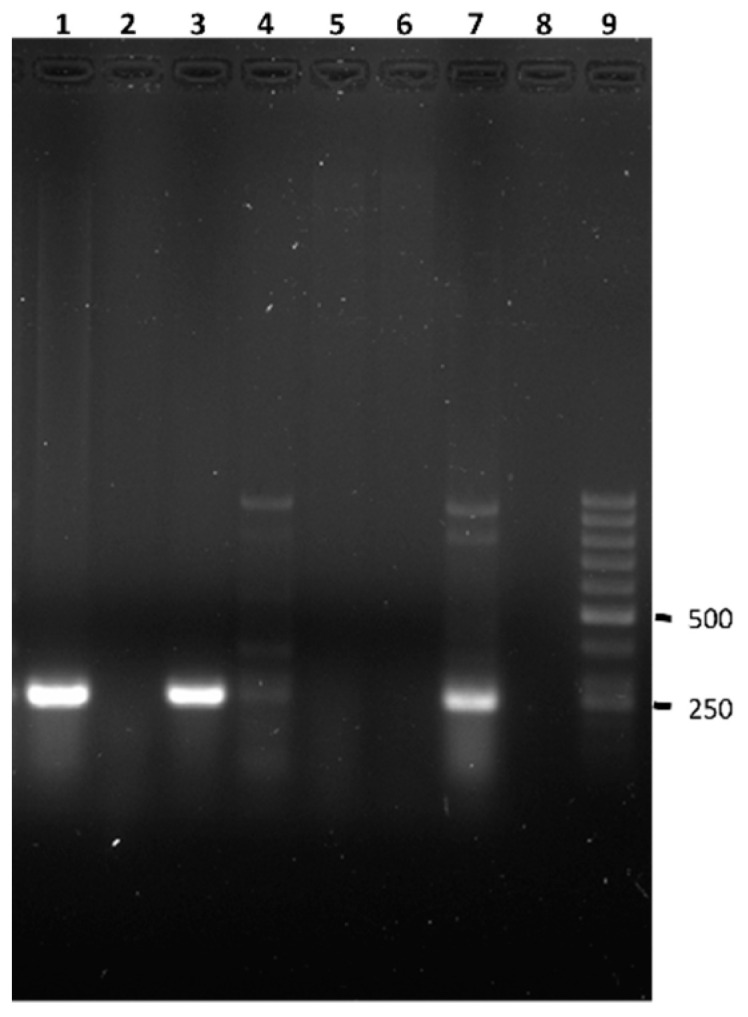
Results of the m-PCR assay for the simultaneous detection of *Pseudomonas* spp. and *P. aeruginosa* from the *Anolis* sp. analyzed. Lane 1 and 3, amplification fragment of the *oprI* gene characteristic of *Pseudomonas* sp. obtained from *Anolis* sp. analyzed in this study. Lane 2, 4, 5, and 6, *Anolis* sp. negative for *Pseudomonas* sp. Lane 7, positive control. Lane 8, control negative. Lane 9, molecular size marker (GeneRuler 50 bp DNA Ladder, Thermo Scientific).

The presence of *Pseudomonas* spp. was investigated in 28 anoles, of which 12 were males, 12 females, and four juveniles. *Pseudomonas* spp. was detected in 18 animals (64.3%) based on the presence of the amplification of the expected 249 bp fragment of the *oprI* gene, present in all members of the genus *Pseudomonas* (Figure 3). Of these positive *Anolis* sp., eight were males (66.7% of the males studied), six females (50.0% of the females studied), and four juveniles (100%). In none of the cases was the amplification fragment of 504 bp of the *oprL* gene obtained and, therefore, none of the isolates corresponded to *P. aeruginosa*. Both weight and length were similar among the individuals with and without *Pseudomonas* sp.

### 3.7. Listeria monocytogenes, Mycobacterium sp., and Yersinia enterocolitica

All individuals investigated were negative for *Listeria monocytogenes*, *Mycobacterium* sp., and *Yersinia enterocolitica*.

### 3.8. Co-Infection and Index of Co-Infection (Ic)

Because *Pseudomonas* sp. were not analyzed in all animals, they were not taken into account in the study of co-infections.

Of the 74 animals analyzed, in 48 individuals (64.9%), at least one of the investigated pathogenic bacteria was detected. Regarding the infection rate, 26 *Anolis* sp. were hosting just one pathogen (35.1%), 19 hosted two pathogens (25.7%), five hosted three (6.8%), and one hosted four pathogens (1.4%) (*C. upsaliensis*, *E. coli* (*stx_1_*), *Vibrio* spp. y *S. lugdunensis*). The most common combination (Table 4) was STEC with the gen *stx_2_* and some species of the *Campylobacter* genus (30.4%), followed by the combination of *Campylobacter* spp. and *S. lugdunensis* (26.1%), STEC that carried the *stx_1_* gen and some *Campylobacter* species (21.7%), and lastly, *S. lugdunensis* and STEC that carried the *stx_2_* gen (17.4%). Finally, in 81.1% of the co-infection cases detected, some of the genes involved in the virulence of STEC were found, while in 64.9%, some *Campylobacter* species was found.

The co-infection index (Ic) for interactions between the different pathogenic bacteria taken into account is presented in Table 4. All the co-infections that presented positive Ic and also the differences between the number of co-infected *Anolis* sp. and the expected numbers were significant (values in bold), suggesting that there is significant association between the bacteria.

According to the data obtained, there is a close association between *Campylobacter* spp. with *S. lugdunensis*, *stx_1_* gene, and *Salmonella* spp., while the presence of the *stx_2_* gene is significantly associated with *Vibrio* spp. (Table 5). Some of the co-infections presented negative Ic, which suggests non-significant associations between the pathogens studied.

## 4. Discussion

### 4.1. Salmonella spp.

The *Salmonella* genus is widely distributed in nature, being found in the gastrointestinal tract of domestic and wild mammals, birds, insects, and reptiles, causing significant disease in both humans and animals [29]. Several studies have shown that reptiles are often infected by different *Salmonella* spp. serovars [30]. The transport of *Salmonella* spp. in the intestinal contents of cold-blooded animals and its importance as a natural reservoir in the epidemiology of *Salmonella* has been recognized in several reports from different geographical areas [31,32,33]. A large number of species, including wild animals found in zoos or laboratories, have been investigated as potential reservoirs for *Salmonella*. Prevalence is variable, although some groups of reptiles, particularly lizards, show higher rate of infection [34,35,36]. *Salmonella* infections in these animals can entail a risk for public health, since they can be a source of infection for the human population living in their vicinity [37,38].

The prevalence of *Salmonella* spp. found in *Anolis* sp. from Tenerife (5.40%) was similar to that detected in the natural populations from Florida (USA) with a prevalence of 7.5% [39], and to the prevalence obtained in other invasive populations of these reptiles established on the main island of Okinawa (2.1%) [40], but lower than that detected in other parts of the world such as Guam (Polynesia, USA) (76.2%) [12] or Chichi Island (Japan) (34.2%) [41]. The low prevalence of these bacteria found in the invasive population of the Canary Islands stands out with the high prevalence found in some endemic lizards, such as *Gallotia stehlini*, with values of 100% [42]. Reptiles do not continually excrete *Salmonella* in their feces, which can present a barrier to identifying infected reptiles if they are subjected to a single bacteriological examination [43]. This fact may have contributed to the lower prevalence found in the population of *Anolis* sp. studied in Tenerife. Another reason could be that not enough time has elapsed since the arrival of these reptiles on the island for the cycle of *Salmonella* infection, that originates in the soil and/or in the feces of other wildlife [12]. The interactions that have to occur between anoles and other infected animals and/or the environment have not occurred frequently enough in the environment where they are found [44].

The absence of the zoonotic species of *Salmonella* sp. investigated in this study does not rule out the potential transmission of these reptiles from other species or serotypes that can potentially be transmitted to humans, which is demonstrated by the detection of other species of the genus in the animals analyzed.

### 4.2. Campylobacter spp.

Bacteria included in the genus *Campylobacter* are common pathogens of great important to public and veterinary health worldwide [45,46], due to their zoonotic potential, wide host range, ability to colonize diverse habitats, and emerging resistance to some of the commonly used antimicrobials [47].

Some *Campylobacter* species can be found in many different host species, such as the *C. jejuni* and *C. coli* species found in this work. Other species are more associated with, or even restricted to, certain hosts such as *C. upsaliensis* in dogs, a species found also in the anoles studied in this work [48].

In reptiles, the most common *Campylobacter* species are *C. iguaniorum* Gilbert et al., 2015 and C. *hyointestinalis* Gebhart et al., 1985; On et al., 1995, and the subspecies *C. fetus* subsp. *fetus* and *C. fetus* subsp. *testudinum* [49]. Other species apart from these have been detected in reptiles that lived near birds in zoos or fed on poultry and most likely were exposed to thermotolerant species of *Campylobacter*, such as *C. coli* and *C. jejuni* [50].

In this work, *C. fetus* was identified and, although the PCR technique used was not able to discriminate at the subspecies level, the *C. fetus* isolates obtained probably correspond to the most common cited in reptiles, *fetus* and/or *testudinum*.

*Campylobacter jejuni* is typically found in mammals and birds, although it has also been isolated from lizards, as in this work. This differential presence could be explained by the optimal temperatures for growth in endotherms and ectotherms. Mammals and birds have a constant body temperature of 37° and 41–42 °C, respectively; these temperatures would favor the growth of *C. jejuni*, whose optimum growth temperature is 37–42 °C [51].

The temperature range of ectothermic vertebrates is 5–46 °C, while the optimum temperature for growth in *C. iguaniorum* and *C. fetus* subsp. *testudinum* is from 20 to 37 °C [52]. This temperature range may be an adaptation that favors the growth of these pathogens in the reptilian host, since the average voluntary temperature for reptiles ranges between 20 and 35 °C [50].

The presence of *C. jejuni*, *C. coli,* and *C. upsaliensis* in the analyzed anoles may be due to these animals sharing the habitat with other animals in which these thermotolerant species are usually found, such as birds and dogs. In fact, it has been shown that, for example, *C. jejuni* can alter some gene expression pathways and adapt to new growth temperatures when exposed to a sudden change in temperature [53], a fact that may explain the development of these thermotolerant species in anoles.

Apart from *C. jejuni*, *C. coli,* and *C. upsaliensis*, species implicated in causing infections in humans, the reptile-associated *C. fetus* species has been also reported, causing infection in humans with underlying disease [54]. *Campylobacter fetus* subsp. *fetus* and *C. fetus* subsp. *testudinum* have been identified as the predominant causes of human campylobacteriosis linked to reptiles. Additionally, *Campylobacter jejuni* has been frequently isolated from squamates and reported as a potential health risk to humans [55].

Reptiles are potentially involved in the horizontal transmission of *Campylobacter* spp. either by cross-contamination through their feces, handling of pets, or, in general, as a result of close interaction with human habitats [56].

For all these reasons, the presence of the *Campylobacter* species identified in the anoles analyzed implies that these animals can be a potential source of infection for people who interact with these animals.

### 4.3. Vibrio spp.

*Vibrio* species are highly related to aquatic ecosystems. This genus contains 66 species [57], of which 12 are pathogenic for humans or have been isolated from clinical samples. Of these species, the ones that stand out for their pathogenicity are *V. cholerae*, *V. parahaemolyticus*, and *V. vulnificus* [58].

Although water constitutes the main reservoir of this etiological agent, *Vibrio* species have been isolated in numerous species of mammals, both domestic and wild, and in birds [59,60]. In non-cholera-endemic areas, strains of choleric and non-cholera (non-cholera-producing) vibrios have been isolated from domestic animals (goats, cows, dogs, and birds) [61].

Although the agent has been isolated from many animal species, their role as reservoirs is unclear [62], although some researchers have considered them to be reservoirs or possible sources of infection for humans [59,61]. The presence of *V. cholerae* in these reptiles makes them a potential source of this bacteria for people who come into contact with them.

### 4.4. E. coli STEC

There are numerous studies on the virulence of *E. coli* isolated from mammals, including humans and birds; however, data on the presence of these bacteria in reptiles are scarce. What has been shown is that the prevalence of *E. coli* in reptiles is low compared to that detected in warm blooded animals [63,64,65,66]. In addition, the existing works have only been focused on investigating the presence of virulence factors in reptiles in captivity [67,68].

Bautista-Trujillo et al. [68] found the presence of virulent strains of *E. coli* in captive green iguanas, with a higher prevalence for the *stx_1_* gene (38.7%) than for *stx_2_* (1.6%) or *eae* genes (3.2%). However, in this study the highest prevalence was detected for the *stx_2_* gene (28.4%), followed by the *stx_1_* (10.8%) and the *eae* genes (5.4%). On the contrary, Dec et al. [69] did not detect any of the genes investigated in this study in pet lizards, as in the case of the captive ocellated lizard (*Timon Lepidus,* Daudin, 1802) in a research center in Spain [67]. The population of *Anolis* studied in this work is located in a landscaped area irrigated with treated wastewater with fecal coliform values between 3.0·10^3^ and 1.0·10^5^ CFU/mL [70], so the presence of *E. coli* is guaranteed. It has been shown that wastewater of human origin, as is the case, and of animal origin, are potential reservoirs of bacteria carrying the *stx_2_* and *stx_1_* genes [71,72], and that the *stx_2_* gene is present with a higher proportion than the *stx_1_* gene [72]. The continuous contact of anoles with purified water may justify the presence of the virulence genes in this population, and the fact that the most prevalent gene is *stx_2_* instead of *stx_1_*, contrary to that which occurs in other reptile populations.

*Stx* toxins represent the key virulence factor for diarrheal disease caused by STEC strains [73]. Both *stx_1_* and *stx_2_* are related to infections in humans, although *stx_2_* toxin is more virulent as it is responsible for causing hemolytic uremic syndrome in humans [73,74]. The intimin adhesin, encoded by the *eae* gene, is involved in the adherence of bacteria to enterocytes [75]. However, STEC strains that lack the latter gene can also cause disease in humans. Although it is not established which combination of markers define a STEC strain to be pathogenic, the presence of the *stx*/*eae* genes is associated with a risk of more serious human disease [76]. Therefore, the high prevalence found for the virulence genes analyzed in this work, especially for the *stx_2_* gene, confirms the role of these reptiles as reservoirs of *E. coli* STEC, and a risk of transmission both for human and the surrounding wildlife.

### 4.5. Staphylococcus sp.

There are few studies on the presence of *Staphylococcus* sp. in wild populations of scaled reptiles, most focusing on the study of skin infections by *S. aureus* [77] or on the detection of species in oropharyngeal and cloacal isolates [78]. There are few works that describe the prevalence of this bacterial genus in the feces of this type of reptiles. The prevalence of *Staphylococcus* sp. found in the *Anolis* population from Tenerife (14.9%) was among those described in feces from populations of other scaly animals with similar eating habits in other parts of the world, such as *Gecko gecko* Linnaeus in Singapore (7.9%) or *Hemidactylus turcicus* Linnaeus, 1758 in Iraq (19.0%) [79]. In the Canary Islands, *Staphylococcus* sp. has been detected in lizards belonging to *Gallotia* Boulenger, 1916 species kept in captivity, with 100% prevalence for *G. intermedia* Barbadillo, Lacoba, Pérez-Mellado, Sancho and Lόpez-Jurado, 1999, and 88.9% for *G. bravoana* Hutterer, 1985 he [80]. The difference between the prevalence found in *Anolis* and in these Canary endemic lizards may be due to the captive condition of the latter, which favors the spread of this and other bacteria.

The detection of *Staphylococcus lugdunensis* as the only species of this genus in the anoles was surprising, since it has not been previously detected in reptiles. This coagulase-negative staphylococci is a commensal organism, found on the skin and mucous membranes of humans and companion animals [81], such as dogs, cats, goats, chinchillas, and guinea pigs [82]. Anoles may be acquiring these bacteria from their closeness to people and pets.

The relationship found between the presence of *S. lugdunensis* with a lower weight of the anoles may be due to the fact that there are species of this bacterial genus that can cause stomatitis in lizards [83]. Early symptoms may be subtle and often overlooked: slight petechia; inappetence, a reluctance to feed or change in food selection; and increased, thickened, ropey, or sheeting saliva [83]. This can lead to weight loss by the animal. The human infections caused by coagulase-negative staphylococci, unlike those caused by *S. aureus*, manifest as less severe or subacute illnesses. The exception to this rule is the species *S. lugdunensis*. The special virulence, microbiological, clinical, and antimicrobial susceptibility characteristics of this species make it unique and different from another negative coagulase. *S. lugdunensis* behaves more like *S. aureus* in many respects, especially its high virulence and its ability to produce suppurative infections [84]. Due to these characteristics, the presence of *S. lugdunensis* in the anoles studied poses a potential risk to the human population that comes into contact with these animals.

### 4.6. Pseudomonas sp.

About 120 species are grouped within *Pseudomonas* genus, all related to humid environments such as water and soil ecosystems, and are infectious for plants, animals, and humans. Within the species of this genus, *P. aeruginosa* is most often associated with human infection, although it is found naturally in the environment [85,86].

*Pseudomonas* spp. can present high pathogenicity under certain conditions and have been associated with high morbidity and mortality in infected animals due to their ability to develop resistance to antibiotics and express multiple virulence factors [87]. In the case of reptiles, the isolation of *Pseudomonas* sp. is frequent in the oral cavity and intestinal tract [78], since they are part of the normal microbiota. However, and mainly in the case of *P. aeruginosa*, they can be opportunistic pathogens with various clinical manifestations in susceptible animals [88]. In the anoles analyzed, apparently, there was no negative effect due to the presence of these bacteria, since there were no differences in weight and length between individuals with and without *Pseudomonas* sp., possibly due to the fact that *P. aeruginosa* was not detected in this study.

It has been found that the prevalence of bacteria of the genus *Pseudomonas* sp. in different types of lizards is highly variable, with values of 6.2% in the case of the wall gecko (*Hemydactilus turcius*) in Iraq [80], 33.3% in *Agama agama* Linnaeus 1758 in Nigeria [89], 25.0% in *Gallotia intermedia,* and 11.1% in *Gallotia bravoana* in the Canary Islands, Spain [80], or 41.7% in *Podarcis* sp. Wagler, 1830 in Italy [90]. In the case of the population of *Anolis* sp. studied, the prevalence of *Pseudomonas* sp. was higher than those in the cited studies, with a value of 64.29%. As in these other works, this bacterial genus was one of the most prevalent with respect to other pathogenic bacteria.

Several studies describe that, in some gecko species not often associated with the soil, where *Pseudomonas* sp. is frequent, hardly any bacterium of this genus is isolated from the intestinal content of these animals [91], or in a small proportion [79]. On the contrary, in the *Anolis* sp. population of Tenerife, the prevalence of *Pseudomonas* sp. is elevated, despite being arboreal animals rarely found on the ground. This could be due to the exposure of these lizards to these bacteria, since the area where they are located is irrigated with treated wastewater with an abundant presence of *Pseudomonas* sp. [92].

As for the other pathogens analyzed, improper handling of these lizards can be a risk to human health, a fact that has been demonstrated by cases of cross-contamination between captive reptiles, such as snakes, and their owners [93]. In the case of the surrounding fauna, especially lizards and geckos, they are not supposed to pose a risk since all reptiles are normal carriers of this type of bacteria.

### 4.7. Listeria monocytogenes

In this study, *L. monocytogenes* was not detected. This is not surprising, since although it has been detected in a wide variety of mammals, few reports of infections in reptiles are available. The first reported case of reptiles was from alligators that had been fed infected pork [94]. Two other cases involved bearded dragons (*Pogona vitticeps* Ahl, 1926) [95,96], being confirmed that one of them was infected by feeding frozen mice. Our study indicates that *Anolis* sp. in Tenerife are not an important reservoir for this bacterium.

### 4.8. Mycobacterium spp.

Many cases of spontaneous mycobacterial infection have been described in a wide variety of reptiles, including lizards, snakes, crocodiles, and turtles [97,98]. Transmission is still poorly understood, but skin lesions or ingestion are generally believed to be the most likely form of infection [99]. Although they are bacteria that are frequently isolated from soils, waters, and plants [100], and are frequent in lizards [101], however, in the population of *Anolis* sp. studied, no bacteria of this genus were detected.

### 4.9. Yersinia enterocolitica

*Yersinia enterocolitica* is widely distributed in the environment. As one of the few intestinal bacteria that can grow at low temperatures, it has a wide host range, including livestock, poultry, rodents, reptiles, and aquatic animals [102]. However, as in the case of some species of giant lizards in the Canary Islands [80], in the *Anolis* sp. studied in this work, this bacterium was not detected, so it seems that it is not frequent in these animals.

### 4.10. General

In the *Anolis* sp. population investigated, the most prevalent pathogenic bacteria were *Pseudomonas* sp., followed by bacteria containing genes *stx_1_*, *stx_2_*, or *eae,* and *Campylobacter* spp. These results are surprising due to the fact that *Salmonella* sp., one of the most prevalent bacteria in reptiles, was one of the least isolated genera.

Most wild animals are usually affected by multiple simultaneous infections caused by various infectious organisms [103], but the relationships between these micro-organisms can vary. As observed in this study, some of them show antagonistic interactions, others positive, and others do not interact. Interpretation of the results can be difficult because many factors, in addition to simple interactions between micro-organisms, can influence the number of co-infections. In addition, the presence of these pathogens in nature can be influenced by a series of factors, such as the presence of microclimates, vegetation, and the density of reptiles. Likewise, the immune responses caused by some micro-organisms can increase the susceptibility to other infectious agents, or vice versa [104] and, therefore, it can be an important factor in co-infection.

The major mechanism of acquiring several bacteria by lizards occurs by a close contact with mothers just after hatching; studies indicated that mothers may transfer microbes to their progeny vertically through different ways depending on their biology [105]. This generation-to-generation transmission may also be occurring in the anole population studied.

Environment is the second source of bacteria; lizards might acquire them horizontally from air, water, and food [106]. Irrigation with purified wastewater from the existing vegetation in the area where the *Anolis* sp. are located may be an important factor that contributes to the transmission of the pathogenic bacteria to these reptiles..

Anoles are arboreal insectivores, actively consuming large numbers of invertebrates and sometimes small vertebrates, such as geckos [107,108,109]. Most of the insects captured by these reptiles serve as a major source of pathogens as these insects often come into extensive contact with human and animal feces and waste [110].

The presence of pathogenic bacteria detected in the anole population studied poses a risk of zoonosis for the population that comes into contact with them, if proper handling is not performed.

## 5. Conclusions

The *Anolis* sp. population introduced to the Canary Islands (Spain) harbors a variety of pathogens of relevance to public health. Considering the zoonotic importance of the majority of the pathogens detected, handling *Anolis* sp. from Tenerife can pose a risk for human health. Among the bacteria detected, enteropathogenic *Escherichia coli*, *Campylobacter jejuni*, *C. fetus*, *Staphylococcus lugdunensis*, *Vibrio cholera,* and *Salmonella* sp. are all recognized species with zoonotic potential; moreover, this is the first reported isolation of *S. lugdunensis* in reptiles.

The anoles can also transmit the pathogenic bacteria to native animals, including endemic species, and/or spread them to the environment, so the possible role in threatening biodiversity should be taking into account.

Considering the public health and veterinary importance of the present results, even by analyzing a limited number of samples, more studies are required, increasing the sample size to better understand the epidemiology of the pathogenic bacteria present in the invasive *Anolis* species in the Canary Islands.

## Figures and Tables

**Table 1:** Percentage of pathogen bacteria isolated from *Anolis* sp. from Tenerife (Canary Islands).

Bacteria Type	No. of Isolates (n)	Prevalence (%) (CI 95%)
*Pseudomonas* spp.	18 (28)	64.3 (45.5–82.1)
*E. coli* (*stx*_1_/*stx*_2_/*eae* genes)	33 (74)	44.6 (38.8–50.3)
*Campylobacter* spp.	24 (74)	32.4 (21.4–42.6)
*Staphylococcus lugdunensis*	11 (74)	14.9 (10.7–19.1)
*Vibrio* spp.	8 (74)	10.8 (3.7–17.8)
*Salmonella* spp.	4 (74)	5.4 (4.4–6.6)
*Listeria monocytogenes*	0 (74)	0.00
*Yersinia enterocolitica*	0 (74)	0.00
*Mycobacterium* spp.	0 (74)	0.00

**Table 2:** Prevalence of *Campylobacter* sp. detected in *Anolis* sp. from Tenerife, Canary Islands.

Campylobacter Species	Positive Males (Prevalence) (CI 95%) n = 25	Positive Females (Prevalence) (CI 95%) n = 45	Positive Juveniles (Prevalence) (CI 95%) n = 4	Total of Positive Individuals (Prevalence) (CI 95%) n = 74
*Campylobacter fetus*	2 (8.0%) (2.6–18.6)	2 (4.4%) (1.6–10.4)	2 (50.00%) (1.0–99.0)	6 (8.1%) (1.9–14.3)
*Campylobacter coli*	0	5 (11.1%) (1.9–20.3)	0	5 (6.8%) (1.0–12.5)
*Campylobacter jejuni*	3 (12.0%) (0.7–24.7)	1 (2.2%) (0.0–6.5)	0	4 (5.4%) (0.2–10.5)
*Campylobacter upsaliensis*	2 (8.0%) (2.6–18.6)	1 (2.2%) (0.0–6.5)	0	3 (4.1%) (0.0–8.5)
*Campylobacter lari*	0	0	0	0

**Table 3:** Prevalence of enteropathogenic *E. coli* detected in *Anolis* sp. from Tenerife, Canary Islands.

Virulence Genes	Positive Males (Prevalence) (CI 95%) n = 25	Positive Females (Prevalence) (CI 95%) n = 45	Positive Juveniles (Prevalence) (CI 95%) n = 4	Total Positive Individuals (Prevalence) (CI 95%) n = 74
*stx*_1_	4 (16.0%) (1.6–30.3)	4 (0.5–17.1)	0	8 (10.8%) (3.7–17.9)
*stx*_2_	6 (24.0%) (7.3–40.7)	14 (17.6–44.6)	1 (25.0%) (0.0–67.4)	21 (28.8%) (18.1–38.7)
*eae*	1 (4.0%) (3.0–11.7)	2 (4.4%) (0.0–5.9)	1 (25.0%) (0.0–67.4)	4 (5.4%) (0.3–10.5)

**Table 4:** Percentage of co-infections of pathogen bacteria isolated from *Anolis* sp. from Tenerife (Canary Islands, Spain) (n = 74).

No. of Individuals	Mixed Infection of Bacteria/Virulence Gen	Percentage %
7	*Campylobacter* spp. + *stx*_2_ gene	30.4
6	*Campylobacter* spp. + *Staphylococcus lugdunensis*	26.1
5	*Campylobacter* spp. + *stx*_1_ gene	21.7
3	*Campylobacter* spp. + *Salmonella* spp.	13.0
3	*stx_2_* gene *+ Vibrio* spp.	13.0
3	*stx*_2_ gene + *Staphylcoccus lugdunensis*	13.0
2	*Campylobacter* spp. + *Vibrio* spp.	8.7
2	*stx_1_* gene *+ Staphylcoccus lugdunensis*	8.7
2	*stx_1_* gene + *stx_2_* gene	8.7
1	*Campylobacter* spp. + *eae* gene	4.4
1	*stx_1_* gene + *Salmonella* spp.	4.4
1	*stx_1_* gene *+ Vibrio* spp.	4.4
1	*Staphylcoccus lugdunensis* + *Vibrio* spp.	4.4

**Table 5:** Index of co-infection (Ic) for the different co-infections obtained from *Anolis* sp.

Mixed Infection of Bacteria/Virulence Gen	Ic	Chi-Square
*Campylobacter* spp. + *stx*_2_ gen	0.28	0.29
***Campylobacter* spp. + *Staphylococcus lugdunensis***	**4.80**	6.32 *
***Campylobacter* spp. + *stx*_1_ gen**	**16.92**	10.2 *
***Campylobacter* spp. + *Salmonella* spp.**	**10.48**	17.6 *
***stx_2_* gen *+ Vibrio* spp.**	**7.80**	7.24 *
*stx*_2_ gen + *Staphylcoccus lugdunensis*	2.86	3.98
*Campylobacter* spp. + *Vibrio* spp.	−1.96	10.2 *
*stx_1_* gen *+ Staphylcoccus lugdunensis*	10.23	0.54
*stx_1_* gen + *stx_2_* gen	−1.00	7.24 *
*Campylobacter* spp. + *eae* gen	−0.93	17.1 *
*stx_1_* gen + *Salmonella* spp.	5.18	1.44
*stx_1_* gen *+ Vibrio* spp.	2.26	0.00
*Staphylcoccus lugdunensis* + *Vibrio* spp.	−1.05	0.52

* and bold type: significant differences between the number of co-infections obtained and expected. * no bold type: non-significant associations between the pathogens studied.

## Data Availability

Data is contained within the article.
